# Locally induced neural stem cells/pluripotent stem cells for *in vivo* cell replacement therapy

**DOI:** 10.1186/1755-7682-1-17

**Published:** 2008-09-24

**Authors:** Ti-Fei Yuan, Oscar Arias-Carrión

**Affiliations:** 1 Department of Anatomy, The University of Hong Kong, Li Kai Shing Faculty of Medicine, 21 Sassoon Road, Hong Kong, PR China; 2 Experimental Neurology, Biomedical Research Center (BMFZ), Philipps University, Hans Meerwein Str, D-35043 Marburg, Germany

## Abstract

Neural stem cells hold the key to innovative new treatments for age-associated degeneration and traumatic injury to the brain and spinal cord. We hypothesized that the *in vivo* induced pluripotent stem cells or neural stem cells through "forced gene expression" can be used to repair damaged brain areas or treat degenerative diseases. Hopefully, these *in vivo* patient-specific stem cells can bring a new avenue for cell replacement therapies.

## Background

One characteristic of many neurodegenerative diseases – which include stroke, brain trauma, spinal cord injury, amyotrophic lateral sclerosis, Huntington's disease, Alzheimer's disease, and Parkinson's disease – is neuronal-cell death. At the moment, few or no effective options are available for their treatments. Neural stem cells hold the key to innovative new treatments for age-associated degeneration and traumatic injury to the brain and spinal cord. In this way, induced pluripotent stem cells (iPSCs) have been successfully produced in both mouse [1-4] and human somatic cells [5,6]. The artificially generated iPSCs were remarkably similar to other naturally isolated stem cells, and thus can give birth to all kinds of cell types, including neurons. The potential of inducing a non-pluripotent cell in order to become a pluripotent stem cell therefore opens a new avenue beyond transplantation in cell-replacement therapies to degenerative diseases. Here we propose that one important application is to produce neural stem cells from differentiated neurons *in vivo* in the central nervous system, and to repair the dysfunctional neural circuits, whether caused by aging, disease or a knife cut.

Neural stem cells have been transplanted into the brain for some time now for therapeutic purpose. The survival and integration of embryonic stem cells or adult stem cells, however, are largely challenged by the immune rejection, and may run into ethical issues. Additionally, the pharmacologically or physiologically enhanced endogenous neurogenesis is limited to hippocampus and subventricular zone, both of which are restricted to specific areas, and are less likely to be helpful in many brain diseased conditions. Thus, if the genetically "forced" cell type transformation can be repeated *in vivo* on nervous system tissues, the newly generated neurons are free from immune rejection, and may have better chances of integration into original circuits.

## Hypothesis

The *in vivo* induced pluripotent stem cells or neural stem cells through "forced gene expression" can be used to repair damaged brain areas or treat degenerative diseases. Several families of genes have been identified and used on induction of iPSCs, such as Oct3/4, Sox, Klf, Myc, Nanog and LIN28. We hypothesize that these genes can also be used in producing *in vivo* iPSCs or induced neural stem cells. Further genes, however, are required to produce fate determined neural cell lineages in a well-controlled manner. One challenge will be balancing the effects of different transcription factors to make these induced stem cells be pluripotent enough to give birth to neurons or glial cells at proper sites. However, slow work can lead to tumor formation.

For cell therapy, it should be noted that teratoma formation is an inherent feature of iPSCs. However the use of non-viral submission may eliminate such risks. As this tumorigenic activity is lost following differentiation, pharmacological approaches and genetic manipulation can be adopted to promote the neural differentiation. Finally, careful physiological evaluations are required to confirm the same function of neurons as if induced from a glia.

## Testing the hypothesis

The first step will be the induction of neurons or glial cells in culture into neural stem cells or pluripotent stem cells, using similar transcription factors that have succeed in skin cells, for example, or to indentify extra transcription factors required. Then either viral transfection or electroporation could be adopted for *in vivo* gene transfer, "inducing" neural cells inside the brain into stem cells. Lastly the therapeutic values of these newly generated neurons have to be tested, to see if they can bring any functional outcomes.

## Implications of the hypothesis

Gene therapy on the brain has been well developed. Viral vectors including lentivirus and retrovirus were widely used, both of which are used in producing the iPSCs [5,6]. Furthermore, the non-viral approaches such as electroporation can achieve good localization as well as high efficiency in both embryo and adult brain [7]. Liposome [8] or nanoparticles [9] based gene submission also offer strong supports for genetic manipulations *in vivo*. Other potential methods that could be used are sonoporation that adopt ultrasound in mediating the gene transfer [10-12]. Theoretically, sonoporation can be applied on any perfused tissue or organ, including deep brain areas; what's more, this non-invasive approach is free of microinjection into the brain and the drugs can be embedded into "blood-brain-barrier-free" bubbles, taken orally or injected intravenously for brain submission. All these methods that submit genes or molecules into specific areas provide eligible avenues in producing iPSCs or neural stem cells locally inside the brain. Hopefully, these *in vivo* patient-specific stem cells can bring a new avenue for cell replacement therapies.

## List of Abbreviations

iPSCs: induced pluripotent stem cells.

## Competing interests

The authors declare that they have no competing interests.

## Authors' contributions

Both authors equally contributed to the manuscript.
